# Long and Short Non-Coding RNAs as Regulators of Hematopoietic Differentiation

**DOI:** 10.3390/ijms140714744

**Published:** 2013-07-15

**Authors:** Franck Morceau, Sébastien Chateauvieux, Anthoula Gaigneaux, Mario Dicato, Marc Diederich

**Affiliations:** 1 Laboratoire de Biologie Moléculaire et Cellulaire du Cancer, Kirchberg Hospital, 9 Rue Edward Steichen, 2540, Luxembourg; E-Mails: franck.morceau@lbmcc.lu (F.M.); sebastien.chateauvieux@lbmcc.lu (S.C.); anthoula.gaigneaux@lbmcc.lu (A.G.); mdicato@gmail.com (M.D.); 2 Department of Pharmacy, College of Pharmacy, Seoul National University, Seoul 151-742, Korea

**Keywords:** ncRNA, miRNA, lncRNA, transcription factors, regulatory network, erythropoiesis, leukemia, lymphoma, differentiation

## Abstract

Genomic analyses estimated that the proportion of the genome encoding proteins corresponds to approximately 1.5%, while at least 66% are transcribed, suggesting that many non-coding DNA-regions generate non-coding RNAs (ncRNAs). The relevance of these ncRNAs in biological, physiological as well as in pathological processes increased over the last two decades with the understanding of their implication in complex regulatory networks. This review particularly focuses on the involvement of two large families of ncRNAs, namely microRNAs (miRNAs) and long non-coding RNAs (lncRNAs) in the regulation of hematopoiesis. To date, miRNAs have been widely studied, leading to a wealth of data about processing, regulation and mechanisms of action and more specifically, their involvement in hematopoietic differentiation. Notably, the interaction of miRNAs with the regulatory network of transcription factors is well documented whereas roles, regulation and mechanisms of lncRNAs remain largely unexplored in hematopoiesis; this review gathers current data about lncRNAs as well as both potential and confirmed roles in normal and pathological hematopoiesis.

## 1. Introduction

**Figure 1:**
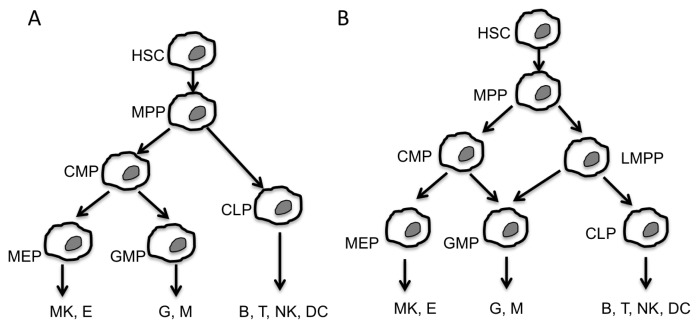
Two models of the hierarchical hematopoiesis process. Hematopoietic cell differentiation proceeds by successive hierarchical maturation steps. (**A**) Pluripotent hematopoietic stem cells (HSC) give rise to multipotential progenitors (MPP) leading to common lymphocyte progenitors (CLP) and common myeloid progenitors (CMP). CLPs directly generate cells of the immune system. CMPs give rise to megakaryocyte-erythroid progenitors (MEP) and granulocyte-macrophage progenitors (GMP); (**B**) The alternative model differs by the involvement of an intermediate lymphoid-primed multipotential progenitor (LMPP) to generate GMP and CMP. Both models lead to the production of differentiated hematopoietic cells (M, monocyte; G, granulocytes; E, Erythrocyte; MK, megakaryocyte; T and B, lymphocytes; NK, natural killers DC, dendritic cells).

Hematopoiesis is the physiological process leading to the production of all circulating blood cells. Hematopoietic stem cells (HSCs) are pluripotent cells with high self-renewal capacity. Asymmetric division is the main approved model for self-renewal and commitment towards a specific differentiation pathway [1], which allows maintaining a steady state HSCs population by preserving hematopoietic homeostasis. As shown in Figure 1, at least two models have been proposed for the hematopoietic hierarchy.

Cell fate decision is regulated by a complex network of extra- and intracellular regulatory factors to ensure HSCs commitment, survival, differentiation as well as maturation depending on physiological requirements. Many cytokines and growth factors activate cell-signaling pathways controlling posttranslational modifications of transcription factors (TF), protein-protein interactions, enzyme activation, binding of proteins to both DNA and RNA, stability of proteins and mRNAs and activation of epigenetic regulators. The ultimate step of this intricate combination of activated and inactivated proteins is the transcription or silencing of genes. To reach this status of differentiated cells, a continuous fine-tuning of genetic programs is essential for the control of the rhythm of cellular divisions, which decreases with cell differentiation, to prevent early cell death of progenitors and precursors as well as to express specific phenotypes. Besides key factors required for self-renewal and commitment of HSCs, complex regulatory networks of TFs mark the cell paths and participate up until their destiny. During hematopoiesis, TF activities depend on interactions between themselves and with cofactors. In the myeloid and lymphoid branches of hematopoiesis, several TFs are differentially involved in divergent pathways. Further TF families are crucial in hematopoietic cell fate decisions. Lichtinger *et al.*, showed that the combination and interdependent regulation of T-cell acute lymphocytic leukemia protein (TAL)1/stem cell leukemia (SCL), Friend leukemia integration (FLI)1 and CCAAT-enhancer-binding protein (C/EBP) is required for the correct temporal expression of lineage specific genes [2]. Malinge *et al.*, reported that the TF Ikaros, which is expressed in erythro-megakaryocyte progenitors [3], blocks terminal megakaryocytic maturation through the inhibition of GATA-1 expression in correlation with target gene inhibition including LIM domain only (Lmo)2. This possibly also involves the inhibition of GATA-1 cofactor Friend of GATA (FOG)-1 by Ikaros. As an alternative mechanism, Notch signaling pathway is also affected by Ikaros-mediated inhibition of megakaryocytic differentiation. Indeed, Ikaros was shown to inhibit the Notch-induced megakaryocytic pattern from hematopoietic progenitors [4]. Lmo2 serves as a bridge between GATA-1 and the SCL complex. Tripic *et al.*, showed that SCL increases GATA-1 transcriptional activity in the murine erythroid cell line G1E-ER4, which displays an inducible GATA-1 construct. Conversely, in the absence of Lmo2, GATA-1 plays a repressive role on target genes in correlation with the absence of SCL complex association.

**Figure 2:**
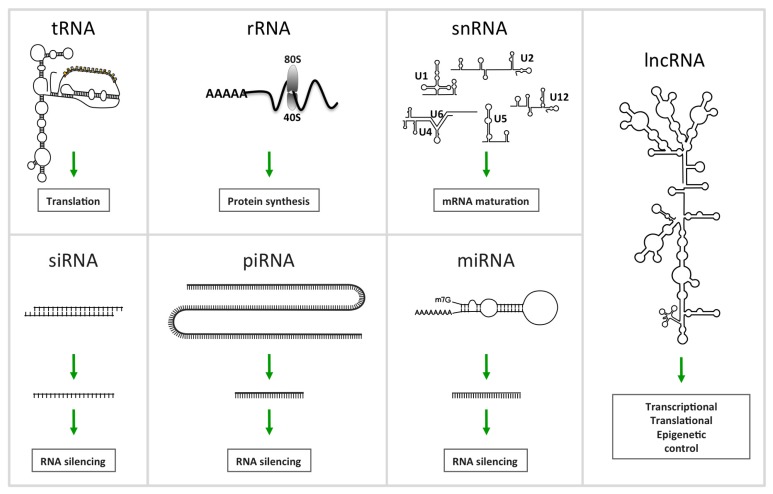
Schematic representation of different non coding RNAs.

Besides, transcription factors play a central role in hematopoietic development, from HSC commitment to terminal differentiation and death. They are differentially and temporally expressed along the differentiation process. Furthermore, an additional level of regulation has joined the network of regulatory factors with the involvement of non-coding RNAs (ncRNAs). This term defines RNA transcripts without protein-coding capacity including constitutively expressed housekeeping small RNAs, ribosomal (rRNAs), transfer (tRNAs), small nuclear (snRNAs), small nucleolar (snoRNAs), transfer-messenger (tmRNAs) and telomerase RNAs. Furthermore, regulatory ncRNAs have been also described including the family of microRNAs (miRNAs or miRs) and the family of larger regulatory ncRNAs, long non-coding (lncRNAs) (Figure 2). This review updates knowledge about regulatory ncRNAs in hematopoiesis by especially focusing on miRNAs as well as the lncRNAs. Particular attention is given to the miRNAs/transcription factors forming regulatory network.

## 2. Non-Coding RNA

Genomic analyses determined that the proportion of the genome coding for proteins corresponds approximately to 1.5%, while at least two thirds are transcribed, suggesting that many non-coding sequences are transcribed into ncRNA. In addition, while protein-coding sequences yet represent a minority of the genome of multicellular organisms, their proportion further declines with increasing complexity of the organism, with a concomitant increase in the amount of non-coding regions in intergenic or intronic sequences [5–7]. A non-coding RNA (ncRNA) or non-protein-coding RNA (npcRNA) is a functional RNA molecule not translated into a protein.

The discovery of RNA interference (RNAi) [8] in *C. elegans* and earlier, the identification of a new class of small RNAs known as miRNAs [9] led to greater attention to ncRNA and their involvement in the regulation of biological processes. The total number of ncRNAs is still unknown, but through transcriptomic and bioinformatic studies, one suspects the existence of several thousands of ncRNAs. A large number of them remain to be fully identified, and the functions of most of them have not yet been validated. NcRNAs are grouped into several RNA families, subclassified according to their function, size, structure and conservation (Figure 2). We thus find the ubiquitous and well-known transfer RNA (tRNA) required as the physical link between the nucleotide sequence of nucleic acids (mRNA) and the amino acid sequence of proteins; The ribosomal RNA (rRNA) as RNA component of the ribosome, and essential for protein synthesis in all living organisms. Small nuclear RNAs (snRNAs), form a class of RNA molecules with an average length of 150 nucleotides localized within the nucleus of eukaryotic cells. Their primary function is pre-mRNA (hnRNA) processing, for which they are always associated with a set of specific proteins and the complexes are referred to as small nuclear ribonucleoproteins (snRNP). A subclass of snRNA is called small nucleolar RNAs (snoRNAs) localized in the nucleolus and implicated in the maturation of RNA molecules by guidance of chemical modifications targeting mainly rRNAs, tRNAs and snRNAs. Piwi-interacting RNAs (piRNAs) form the largest class of small non-coding RNA molecules expressed in animal cells and form the third class of small RNA silencers. They form RNA-protein complexes by interacting with Piwi proteins and are required for both epigenetic and post-transcriptional gene silencing of retrotransposons and other genetic elements in germ line cells, particularly during spermatogenesis. Small Interfering RNAs or silencing RNAs (siRNAs) are a class of short double stranded RNA molecules (20–25 bp) playing a role in the RNA interference (RNAi) pathway. They modulate expression of specific genes by interfering with RNA translation by complementary nucleotide sequences. Post-transcriptional activity of siRNAs was first discovered in plants [10], and their possible use in mammalian cells was rapidly shown [11], evidencing the potential as a biomedical research tool. Interference induced by siRNAs share a large part of the signaling pathways used by miRNA naturally present in mammalian cells. The presence of siRNA encoded by the human genome has never been shown. Extracellular or exosomal RNA (exRNA) designate RNA species present in body fluids (venous blood, saliva, breast milk, urine, semen, menstrual blood or vaginal fluid) composed by mRNA, tRNA, miRNA, siRNA and lncRNA, generally enclosed within vesicular bodies preventing their digestion. Biochemical evidence supports the idea that exRNA uptake is a common process, suggesting new pathways for intercellular communication. The relative abundance of certain exRNAs can be correlated to cellular signaling or specific disease states. Recently a study characterized the population of RNA in human plasma by deep sequencing, and demonstrated that most abundant RNAs are miRNAs (42.32%), followed by ribosomal RNAs (9.16% of all mapable counts), long non-coding RNAs (3.36%), piwi-interacting RNAs (1.31%), transfer RNAs (1.24%), small nuclear RNAs (0.18%), and small nucleolar RNAs (0.01%) [12].

Finally, micro RNAs (miRNAs) and long noncoding RNAs (lncRNAs) form the two families of ncRNA more widely described in our review.

### 2.1. MiR and Hematopoiesis

#### 2.1.1. MiRNAs, a Family of Regulatory ncRNAs

While the first small endogenous ncRNAs (*lin-4/let-7*) were described in *C. elegans* in 1993 [13], these RNAs became by now a large family of regulatory ncRNAs referred as microRNAs (miRNAs) [14–16]. These single stranded RNAs are characterized by their size of about 17–25 nucleotides and are highly conserved during evolution [14]. Mature and functional miRNAs result from a multistage process and their expression leads to post-transcriptional silencing of target genes through epigenetic regulatory functions by repressing mRNA translation [17]. Therefore miRNAs are also involved in a complex cellular network of gene regulation. On one hand, most miRNAs are able to target several mRNAs while a specific mRNA can be targeted by several miRNAs [18,19]. On the other hand, miRNAs target TF mRNAs and in turn TFs regulate miRNA gene expression. Expression of miRNAs is then tightly regulated at both transcriptional and post-transcriptional levels. Primary miRNAs (pri-miRNA) result from the transcription of genes requiring RNA polymerase II or III, and critical epigenetic regulation steps [20]. As for TFs, miRNAs display tissue and developmental specificities. In correlation with their spatial and temporal expression, miRNAs contribute to the regulation of embryogenesis, cell proliferation, differentiation and death [21]. Therefore, deregulation of miRNA expression, caused by genetic alterations, transcriptional or processing failures is incriminated in the development of many human diseases, including cancer.

Transcriptional regulation of miRNAs is not yet fully understood while processing of the transcript in mature miRNA has been described in detail [20,22]. Briefly, in the canonical miRNA biosynthetic pathway, the transcription of miRNA genes by RNA polymerase II (or III for miRNAs encoded within Alu repeat sequences) [23] generates a pri-miRNA displaying hairpin structures. In fact, pri-miRNA transcripts can be organized in clusters encoding multiple miRNA sequences [24]. In the nucleus, pri-miRNAs are then cleaved between the hairpin structures to generate pre-miRNAs of 60 to 110 nt. This cleavage is performed by the nuclear endoribonuclease Drosha (RNaseIII) and DiGeorge critical region 8 (DGCR8) cofactor (Pasha in Drosophila)-forming complex. Pre-miRNAs are then exported to the cytoplasm by Exportin-5 in a Ran-GTP dependent manner, where they undergo further processing. Cytoplasmic endonuclease RNase III Dicer associated to the transactivating response RNA-binding protein (TRBP) and the protein activator of PKR (PACT), cleave the stem loop of the hairpin structure to form an asymmetric RNA duplex of about 22 nt. Double strand RNA interacts with Argonaute within the RNA-induced silencing complex (RISC) in order to select the guide strand matching with the 3′-untranslated region (UTR) of the target mRNA, thus forming the miRISC. The complementary “passenger” strand of the duplex is released and degraded. The guide strand allows the RISC-mediated inhibition of translation of the target mRNA as the main mechanism of action. Nevertheless, destabilization of target mRNAs through the accelerated deadenylation was reported as an alternative mechanism for miRNAs action [25,26]. Recently, an additional mode of action was proposed in which endogenous miRNAs act at the transcriptional level to silence genes, involving chromatin remodeling complexes [27].

#### 2.1.2. MiRNAs Regulate the Regulatory Network during Hematopoiesis

**Figure 3:**
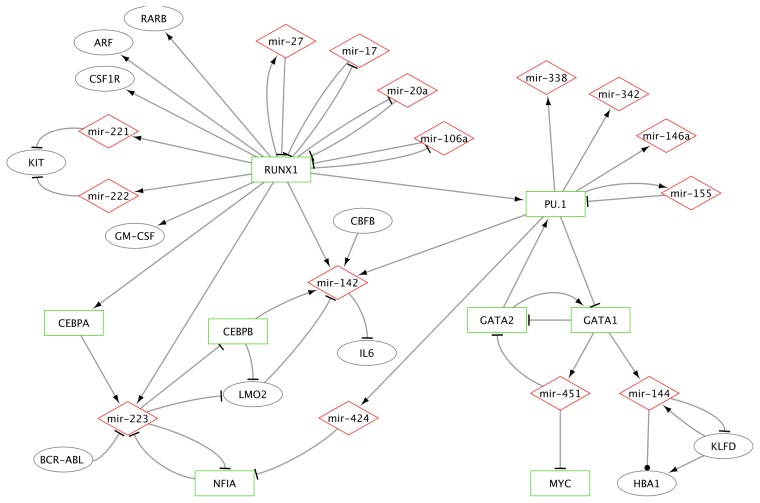
Partial representation of the network involving microRNAs (mir) and regulatory proteins in hematopoiesis. Network was built using Cytoscape 2.8.2 software [36]. Transcription factors are represented as green rectangles, miRNAs as red diamonds, and other proteins as grey ellipses. Arrows depict relationships: arrows (activation), T (inhibition), circle (undetermined/binding). RARB, Retinoic acid receptor B, ARF, Alternate Reading Frame, CSF1R, colony-stimulating factor 1 receptor; BCR-ABL, breakpoint cluster region-Abelson; CBFB, Core-binding factor subunit beta; IL6, interleukine 6; LMO2, LIM domain only 2; HBA1, hemoglobin A1; KLFD, Krüppel like factor D; RUNX1, Runt-related transcription factor 1; CEBPA, CCAAT/enhancer-binding protein alpha; CEBPB, CCAAT/enhancer-binding protein beta; GM-CSF, granulocyte macrophage-colony stimulating factor; NFIA, nuclear factor I-A.

The crucial role of miRNAs in the regulation of specific steps of hematopoiesis is now well-documented based on the modulation of their expression during HSC differentiation processes. Furthermore, in the hematopoietic system, numerous miRNAs were described to affect translation or stability of mRNAs including mRNAs encoding TFs. Thus, interplay of TFs and miRNAs are considered essential regulators of gene expression in hematopoiesis (Figure 3).

##### 2.1.2.1. MiR-223 Is a Key Hematopoietic miRNA

One of the most extensively studied miRNA in hematopoiesis is miR-223 also described as the “fine-tuner” of granulocytic differentiation, maturation and function while its expression decreases during monocytic, erythroid [28] and mast-cell differentiation [29–32]. Nevertheless, up-regulation of miR-223 was observed by Lu *et al.* [33] and Choong *et al.*, during EPO-mediated erythroid differentiation of human CD34^+^ HSPCs [34], whereas its expression was down-regulated in K562 cells. Finally, a functional study revealed a role for miR-223 in erythroid differentiation [35]. According to the various expression levels in different types of hematopoietic cells, miR-223 is linked to several major specific transcription factors.

Expression of miR-223 is associated with granulocyte differentiation and was shown to increase in retinoic acid (RA)-induced granulocytic differentiation of leukemic cells. C/EBPα is a key factor for the commitment of hematopoietic cells towards the granulocytic lineage and its RA-mediated upregulation was anticipating miR-223 activation in NB4 and HL60 promyelocytic cells, suggesting a role for this TF in the transcriptional regulation of miR-223. In correlation, two putative C/EBPα binding sites were identified upstream of the region encoding the pre-miR-223 sequence. Results were confirmed by transfection of expression constructs, which demonstrated the region containing the C/EBPα binding sites to be required for miR-223 responsiveness to RA. Moreover, chromatin immunoprecipitation (ChiP) assays showed that C/EBPα binds *in vivo* to miR-223 promoter. Besides C/EBPα, nuclear factor I-A (NFI-A) binding site was described in the miR-223 promoter [29], overlapping one of the two C/EBPα sites. This TF is also involved in the regulation of miR-223 expression and maintains its basal level in undifferentiated cells while C/EBPα over-expression substitutes NFI-A in RA-induced granulocytic differentiation eventually leading to miR-223 up-regulation. Furthermore, NFI-A is targeted by miR-223. Altogether, granulocytic differentiation correlates well with miR-223-mediated inhibition of NFI-A mRNA translation, therefore preventing competition with C/EBPα [29].

Interestingly, a recent study reported an alternative mechanism for NFI-A regulation by endogenous miR-223. Authors demonstrated the ability of miR-223 to affect transcription of NFI-A gene in addition to a post-transcriptional effect [27]. Authors first observed that nuclear import of miR-223 increased during granulocytic differentiation of myeloid precursors. Using confocal microscopy, results showed that the nuclear compartmentalization of miR-223 was increased in RA treated HL60 cells and primary acute promyelocytic leukemia (APL) blasts cells. ChIP assays performed in HL60 cells revealed *in vivo* presence of miR-223 at complementary sequences of the NFI-A promoter. Moreover, mutation of miR-223 binding sites abolished miR-223-mediated repression of NFI-A promoter reporter constructs [27]. Beyond the increasing variety of miRNA function in hematopoiesis, these findings reveal a novel mechanism of transcriptional control.

According to the pivotal role of miR-223 in hematopoiesis, its deregulation contributes to leukemia development. Notably, it was recently established that the fusion oncokinase breakpoint cluster region (Bcr)-Abelson (Abl) repressed miR-223 expression in chronic myeloid leukemia (CML). miR-223 expression was also significantly decreased in B cell lymphoproliferative disorders [37] and could serve as a prognostic factor for CLL and CML patients.

Transcription factors Satb1 and Runt-related transcription factor 1 (RUNX1) or acute myeloid leukemia 1 protein (AML1) were recently described as central regulators of the early stage of HSCs commitment and self-renewal. Many publications report RUNX1 as an essential TF for hematopoiesis initiation at both adult and embryonic levels [38–40]. In mice embryos, expression of this TF has been localized in all sites giving rise to hematopoietic cells. Hemogenic sites such as intra-aortic hematopoietic clusters are absent in RUNX1^−/−^ embryos [41] and the hematopoietic defect in RUNX1^−/−^ embryonic stem cells was rescued by ectopic expression of RUNX1 in mice [39,42]. Recently, it was also shown that the isoform RUNX1a enhances hematopoietic lineage commitment from human embryonic stem cells [43]. RUNX1 is required for both embryonic and post-natal hematopoiesis. It is also involved in the control of genes essential for myeloid differentiation. A connection between this TF and several miRNAs related to specific myeloid genes has been reported. Notably, RUNX1 also regulates miR-223 gene transcription in myeloid precursors, maintaining chromatin in a transcriptionally active state. Consequently, the fusion protein RUNX1/ETO (AML1/ETO), in which RUNX1 is inactivated, specifically triggers transcriptional silencing of miR-223 [44]. RUNX1/ETO, which causes acute myeloid leukemia (AML), acts as a dominant-negative repressor of RUNX1 target genes [45], including colony-stimulating factor 1 receptor (*CSF1R/*c-fms), GM-CSF [46,47], tumor suppressor p14(ARF) and the retinoic acid receptor β (RARB) [44].

Although up-regulation of miR-223 was correlated to decreased NFI-A expression and granulocytic differentiation, regulation of specific myeloid genes has not been yet established. Nevertheless, miR-223 together with constitutively expressed miR-142 was shown to decrease proliferation required for myeloid differentiation [48]. It is well established that miR-142 is implicated in the regulation of T cell function and development and B cell lymphoma.

##### 2.1.2.2. MiRs-223/142/155 and Specific TFs as a Miniature Regulatory Network

Besides targeting NFI-A, miR-223 also regulates Lmo2-L/-S isoforms and C/EBPβ expression in myeloid cells. A miR-223-mediated decrease of cell proliferation occurred through a miR223-C/EBPβ-Lmo2-miR142 pathway. This model is based on the findings that miR-223 targets C/EBPβ as well as Lmo2 mRNAs and that C/EBPβ regulates transcription of Lmo2, which is then down-regulated at both transcriptional and posttranscriptional levels. This leads to the expression of miR-142 and cell proliferation attenuation since Lmo2 negatively regulates miR-142 gene transcription [48]. Lmo2 is also involved in erythroid-specific gene expression [49] through the formation of a DNA binding complex involving also TAL1, E2A, Ldb/LNI-1 and GATA1 [50]. In agreement with the regulation of Lmo2 expression by miR-223, it has been reported that forced expression of miR-223 inhibited erythroid development of CD34^+^ HSCs [35]. Altogether these results hint for a negative role of this miRNA in erythropoiesis whereas is positive in myeloid differentiation. Similarly, NFI-A up-regulation promotes erythropoiesis while its silencing promotes granulopoiesis in correlation with miR-223 modulation [29,51,52].

Besides Lmo2, Sun *et al.*, recently demonstrated that PU.1, C/EBPβ RUNX1 and the co-factor CBFβ also regulated miR-142 gene transcription. Specific binding sites for these three TFs exist in the miR-142 gene promoter with PU.1 acting predominantly. Indeed, C/EBPβ and RUNX1 alone led to miR-142 deficiency. However, miR-142 expression levels within hematopoietic cells depend on different combinations of PU.1 together with C/EBPβ RUNX1 [53]. By regulating PU.1 expression, miR-155, another relevant miRNA in hematopoiesis, is involved in miR-142 gene transcription. miR-155 targets PU.1 mRNA triggering its repression and consequently the reduction of miR-142 production. A good illustration of the interrelationship between miR-155, PU.1 and miR-142 is the pathway leading to IL-6 expression. Lipopolysaccharide (LPS) stimulates Toll-like receptor 4 (TLR4), which induces miR-155 expression, and down-regulation of PU.1, subsequently leading to decreased miR-142 expression and thus inducing expression of its target, IL-6. This regulatory mechanism is relevant for IL-6–mediated immunological processes and also the functions of miR-142 in the lymphoid system [53].

##### 2.1.2.3. RUNX1 Connection with miRs-222/221, 17-5p, 20a, 106a and 27a

RUNX1 is implicated in the control of miR-222/221 gene cluster transcription as demonstrated by ChiP and luciferase reporter gene assays in U937 monocytic leukemia cells. Indeed, RUNX1 binds to two regions containing RUNX1-consensus sequences in the miR-222/221 gene promoter displaying four consensus sequences. Moreover, RUNX1 dose-dependently activates miR-222/221 promoter in the reporter gene constructs [54]. Expression of miR-222/221 was increased during GM-CSF-mediated myeloid differentiation of normal bone marrow CD133^+^ stem progenitor cells. These results correlate with the down-regulation of the stem cell factor (SCF) receptor KIT expression whose mRNA is targeted by miR-222/221. Conversely, the expression of miR-222/221 and miR-223 was lower in leukemia cells expressing RUNX1 fusion oncoproteins, in correlation with higher levels of KIT oncogene expression and inhibition of myeloid differentiation [54].

On the other side, RUNX1 binds to the promoters of miRNA 17-5p–92 and 106a–92 gene clusters leading to the inhibition of miRNA 17-5p, 20a and 106a transcription. These miRNAs are upregulated in undifferentiated CD34+ hematopoietic stem/progenitor cells (HSPCs) while their expression decreases during monocytic differentiation and maturation. This down-regulation is correlated to the increasingly expressed RUNX1 protein in monocytic cells. In turn, miRNAs 17-5p, 20a and 106a inhibit RUNX1 translation by interacting with the 3′ UTR generating a regulatory loop. RUNX1 was also shown to act as a regulator of miR-27 transcription, which is involved in erythro-megakaryocytic and granulocytic differentiation pathways. Generating a negative regulatory loop, miR-27 targets RUNX1 mRNA. This leads to RUNX1 down-regulation allowing granulocytic differentiation of myeloblasts [55]. Similarly, TPA (12-o-tetradecanoylphorbol-13-acetate)-induced megakaryocytic differentiation of K562 cells was concomitant with increased miR-27a expression and RUNX1 down-regulation [56]. RUNX1 appears thus as a crucial transcription factor in hematopoiesis, given its expression in the different cell lineages and its ability to directly regulate a wide panel of miRNAs as well as TFs including C/EBP and PU.1, which in turn target promoters of miRNA genes [57].

##### 2.1.2.4. MiR-146a and miR-155 Genes Are Regulated by PU.1

During differentiation progress, TFs integrate the extensive regulatory network insuring hematopoietic homeostasis. GATA-1 and GATA-2 are the major TFs regulating erythro-megakaryocytic pathways whereas PU.1 and C/EBP control myelo-lymphoid differentiation. Nevertheless, GATA-1 and PU.1 proteins physically interact and inhibit each other. PU.1, a member of the Ets(E-twenty six) TF family, is involved in differentiation from HSCs to multipotent progenitors at different levels [58]. PU.1 is required for myeloid and lymphoid [59] differentiation and plays a role in cell fate decision. Its expression levels determine the fate of early T-cell progenitors since over-expression of PU.1 was shown to reorient differentiation to the myeloid lineage [60–63] depending on Notch. In the absence of Notch signaling, PU.1 promotes the myeloid pathway, whereas activation of Notch signaling is observed in T-cell lineage pathways. Del Real *et al.*, demonstrated that in absence of Notch signaling, PU.1 regulates expression of TF genes essential for T-cells, including Myb, Tcf7 and Gata3.

PU.1 also plays a pivotal role in lympho-myeloid development through its ability to regulate transcription of miRNA genes as reported by Ghani *et al.* These authors highlighted transcriptional regulation of miR-146a, miR-342, miR-338 and miR-155 genes by PU.1. Results revealed that miR-146a was the most robustly PU.1-induced miRNA. miR-146a and miR-155 up-regulation was independent of *de novo* protein synthesis suggesting that PU.1 is able to regulate transcription of these miRNA genes. Conversely, miR-342 and miR-338 regulation could require cooperative factors linked to PU.1 [64]. Authors also showed that miR-146a directed selective differentiation of HSCs into peritoneal macrophages during adult hematopoiesis and concluded that PU.1 temporally controls the expression of miRNAs required for correct HSCs differentiation.

PU.1 up-regulation was also shown to control human monocyte-macrophage differentiation through the activation of miR-424 [65]. The authors validated NFI-A mRNA as a true functional target of miR-424. Similarly to the C/EBPα-miR223-NFI-A regulatory circuit that regulates granulopoiesis, PU.1-miR-424-NFI-A regulates monocytic differentiation.

##### 2.1.2.5. Erythroid Specific Expression of miRs-144 and 451

GATA factors play a central role in the network of hematopoiesis regulation through interactions with co-factors. Predominant expression of GATA-1 results in the erythroid differentiation. Moreover, PU.1 activity is also involved in the lineage-specific fate decisions of erythroid or myeloid differentiation through its physical interaction with GATA-1 leading to reciprocal inhibition of their transcriptional activities. Especially, suppression of GATA1 activity by PU.1 leads to a shift in cell fate towards the myeloid-lymphoid lineages [66,67]. Upon myeloid differentiation, PU.1 is overexpressed in correlation with GATA-1 and −2 down-regulation. Using the G1ME hematopoietic cell line derived from *in vitro* differentiation of murine GATA-1^−^ embryonic stem cells (ESC) [68], Chou *et al.*, demonstrated that GATA factors act sequentially to control lineage determination during hematopoiesis, through modulation of repressive effects at key regulatory elements of the PU.1 gene [69]. So far, no link between PU.1 expression and the production of miRNAs regulated by GATA-1 has been demonstrated. GATA-1 was shown to regulate *miR-144/451* gene transcription. GATA-1 up-regulation induced *miR-144* and *miR-451* in G1E cells as well as in human CD34^+^ cells and murine erythroleukemia (MEL) cells in correlation with erythroid maturation [70]. The crucial role of miRs-451/144 in physiological erythropoiesis was also demonstrated in zebrafish and mouse models [71,72]. Expression of *miR-451* is restricted to the erythroid lineage and its induced silencing affected neither megakaryopoiesis nor granulopoiesis in zebrafish embryos. GATA-2, which is able to bind the *miR-144/451* locus in the absence of GATA-1, does not activate transcription. A couple of predicted miR-144 and miR-451 target genes, that were down-regulated after GATA-1 activation, were reported [70]. Interestingly, proliferation regulator c-myc, whose overexpression inhibits erythropoiesis, emerged as a predicted target gene of miR-451. Furthermore, it was shown in zebrafish that GATA-2 3′UTR was targeted by miR-451 but not by miR-144 [72]. *GATA-2* is known to preserve the immaturity of hematopoietic precursor cells [73] and induces overexpression of GATA-1, which in turn triggers GATA-2 down-regulation as a negative regulatory feedback loop, required for normal erythropoiesis. Considering that GATA-2 positively regulates PU.1 gene expression, it seems plausible that miR-451 could play a role in the GATA-1/GATA-2 balance and PU.1 down-regulation to warrant erythroid maturation according to a GATA-1 > miR451 > GATA-2 > PU.1 axis. Besides, this would be in agreement with decreased miR-155 expression observed during erythropoiesis since PU.1 regulates gene expression of this miRNA [31,74]. Mir-144 was reported to selectively regulate embryonic α-globin gene expression during primitive erythropoiesis in zebrafish through negative feedback regulation involving erythroid-specific Krüppel-like (KLF)-D TF, which selectively binds to promoters of both α-globin and miR-144 genes to activate their transcriptions [38,75].

Except for miRs-144/451 that are erythroid-specific miRNAs, most miRNAs are ubiquitously expressed at different stages of hematopoietic differentiation in correlation with TF expression and activities involved in the regulation of lineage specific genes.

### 2.2. LncRNA and Hematopoietic Lineage

#### 2.2.1. LncRNA

Among all sub-categories of ncRNAs, long non-coding RNAs (lncRNAs) constitute the most recent and least characterized family. LncRNAs include all ncRNA larger than 200 nucleotides and not yet categorized in one of the other RNA families. In contrast to small ncRNAs, which are highly conserved among species and which are involved in transcriptional and posttranscriptional gene silencing, lncRNAs are poorly conserved [76] and their modes of regulation are diverse and not yet totally elucidated. The process of lncRNA transcription and maturation is similar to that of mRNA even if their genes are not subject to the same histone modifications (H3K4me3 and H3K36me). As for mRNA, lncRNAs are transcribed by RNA polymerase II. Processing of lncRNA involves 3′ poly(A) tailing and 5′-end capping as well as splicing. LncRNAs have small open reading frames without any protein-coding potential, but can be sometimes be associated with ribosomes in the cytoplasm, suggesting an additional role in mRNA metabolism.

Analysis of the human genome and transcriptome estimated that there are about 23,000 lncRNAs, comparable to the number of protein-coding RNAs and greatly exceeding the number of miRNAs (close to 2000) [77,78]. LncRNAs sequences are spread over the entire genome and can be found on all chromosomes [79]. During the last decade, the number of studies on this type of RNA has been increasing, but the general knowledge remains low given the large number of existing lncRNAs. However, it has already been shown that these lncRNAs are tissue specific and differently expressed under both normal and pathological conditions, implying that they may play important biological roles. Indeed they can regulate biological processes, including cell division, survival, and differentiation, as well as some processes related to cancer development. The first schemes of control or mechanism of action were also recently revealed. Several long ncRNA regulate gene expression by modifying chromatin structure. Different studies demonstrate a wide diversity of mechanisms by which a lncRNA regulate chromatin of a single promoter, a gene cluster, or an entire chromosome [6], in order to activate or silence genes in *cis* or in *trans* [80]. Until now, only a small number of lncRNAs were identified and fully characterized.

LncRNAs play different roles in transcription [81]. For example MALAT1 can regulate preRNA splicing by influencing distribution and phosphorylation of the serine/arginine (SR) splicing factor [82]. They can also increase mRNA stability by interaction with exonuclease XRN1, as shown in a recent study of the Moon [83]. Perfect hybridization between mRNA and lncRNA is another system to stabilize RNA: BACE1-AS fully hybridized to exon 6 of BACE1, which presents a binding site for miRNA-485-5p, thus leading to a competition between lncRNA and miRNA for this site [84]. On the opposite, it has also been shown that these ncRNAs can decrease mRNA stability by promoting degradation of mRNA [85]. LncRNA involvement in the regulation of translation was initially suggested by the observation of an association between lncRNA and ribosomes, even though lncRNA are not translated. This kind of regulation seems to be negative in some cases, for example when lncRNA-p21 annealed imperfectly but throughout the coding sequence of the mRNA of β-catenin or JunB, blocking their translation [86]. Positive regulation was also reported, for example in the case of lncRNA AS-Uchl1 that promoted polysome formation around Uchl1 mRNA [87]. In a less specific way, lncRNA BC1 was shown to inhibit the whole process of translation by preventing the assembly of translation initiation complex in neurons [88].

##### 2.2.1.1. Expression and Regulation of lncRNA

As mentioned previously, lncRNA are tissue specific and differently expressed under normal and pathological conditions, implicating that their transcripts are regulated. While information about this regulation is constantly expending, the way lncRNA abundance is controlled is still widely unknown. Intronic sequences represent 30% of the human genome and represent the major source for lncRNAs (intronic lncRNAs). In this case, regulation of non-coding RNA expression is usually linked to host gene expression. LncRNA sequence can also be independent of genes and its expression is then under promoter dependence. This second subcategory is commonly called Long Intergenic Non-Coding RNA (lincRNA). In addition to their regulation at a transcriptional level, lncRNAs can also be regulated post-transcriptionally [81]. A study about the overall evaluation of ncRNA half-life (involving about 800 transcripts) in mouse neuronal cells showed that all lncRNA differed in stability with a minority of “unstable” lncRNA. Stability appears to depend on localization (intronic lncRNA have a lower stability compared to lincRNA), splicing (spliced non-coding transcripts are more stable than the non-spliced ones), and finally subcellular localization (nuclear lncRNA may have shorter half-lifes compared to cytoplasmic ones) [89]. Most of the lncRNAs are stabilized with a poly(A) tail similar to mRNAs. Another type of stabilization involves a triple helix at the 3′ end of lncRNAs protecting against 3′→5′ exonucleolytic cleavage. This structure was recently shown for lncRNA-MALAT1 in multiple endocrine neoplasia β [90,91].

If lncRNAs present differential stability depending on their location or their origin, stability can also be modulated, thereby altering the turnover of these regulators. It has been recently shown that lincRNA-p21 could be destabilized by human antigen R (HuR)/Argonaute (Ago)-2 complex as well as by the miR Let-7b. Silencing of HuR or Ago2 increases lincRNA-p21 stability, whereas overexpression of let-7b induces destabilization. Degradation of RNA requires decapping and deadenylation, and these aspects of lncRNA degradation are still not well known and need to be further elucidated in order to improve the understanding of lncRNA abundance control [86].

##### 2.2.1.2. Mechanisms

**Figure 4:**
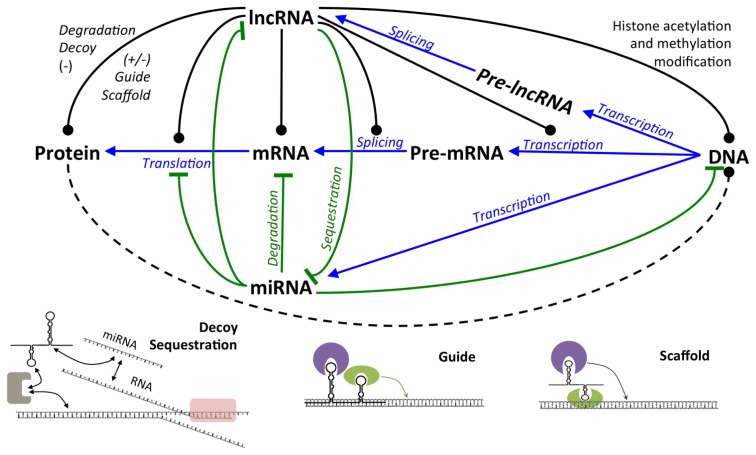
Scheme of regulations involving lncRNAs and miRNAs, influencing transcription, maturation or translation processes and example of interaction between lncRNAs, DNA, proteins and miRNAs. Blue arrows correspond to physiological maturation of RNA. Green lines correspond to inhibition processes and dotted black lines correspond to variable regulations (positive or negative depending on the mechanism). (−) Negative effect; (+/−) negative or positive effect.

Unlike miRNAs, which follow a well-established mechanism of action, causing inhibition of translation or degradation of mRNA, lncRNAs act almost as diversely as proteins (Figure 4). LncRNAs can interact with proteins as well as with RNA or DNA. Interaction of lncRNA with other RNA occurs through total or partial hybridization of complementary sequences. This mechanism is common for anti-sense lncRNAs such as p15AS. It was recently published that interaction of lncRNA with miRNA happens with a preferential hybridization of the miRNA to the 3′ end of the lncRNA [92]. RNA-RNA interaction can lead to translational regulation, but also to splicing or to inhibition of RNA function. LncRNA-DNA interaction can occur similarly to a RNA-RNA interaction, by the sequence complementarily forming a RNA-DNA duplex, or in a more complex configuration such as the hybridization of lncRNA and the dihydrofolate reductase (DHFR) promoter that forms a DNA-DNA-RNA triplex [93], leading to sequence specific transcriptional repression [94]. DNA-RNA interactions recruit DNA regulating factors that can affect transcription, histone or DNA modifications including methylation or acetylation. Finally, the ability of lncRNA to form secondary and tertiary structures allows them to establish complex interactions enabling them to catch and sequester proteins as well as to join distant areas.

In an effort to summarize the different modes of regulation involving lncRNAs, Wang and Chang propose four classes or archetypes of lncRNA regulation [95], further summarized in the recent publication of Da Sacco *et al.* [96]. LncRNAs usually follow several modes of action, and so most of them belong to several of these archetypes.

**Signal**: Expression of these lncRNA can be used as a marker of intracellular signaling or response to stimuli, as it can induce responses *via* transcription of RNA without translation or posttranslational modifications. More a tool than a mechanism, these lncRNAs essentially serve as biomarkers. This archetype therefore concerns all lncRNA with a strong relationship in spatiotemporal response, such as rapid reaction to temperature changes regulated by lncRNAs COLDAIR and COOLAIR [97,98] or imprinting controlled by Xist.

**Decoy**: In molecular decoy, lncRNA compete with another nucleotide sequence or structure for the binding of a TF, chromatin modifier, or other regulatory factor such as miRNA thereby preventing the miRNA to inhibit translation of their targets [99,100]. These lncRNAs are therefore considered negative regulators of effectors.

**Guide**: The third archetype is the guide RNA, which can be described as a connection between lncRNAs and proteins, further guided by the lncRNA to a target. This pattern of regulation stems from the observation that lncRNAs are able to induce changes in gene expression in *cis* (neighboring) or *trans* (distant) genes. Target proteins recognized by the lncRNA may be repressor or activator complexes, or TFs, with the final goal of controlling the expression of a target gene, causing changes in the epigenome, whether the control takes place in *cis* or in *trans*.

**Scaffold** (protein linker): In the fourth archetype proposed by Wang *et al.*, the lncRNA becomes a docking structure for regulatory proteins, in order to assure accurate assembly of ribonucleoprotein complexes. In many biological signaling processes, this control feature is essential for specificity and dynamics of molecular interactions and signaling events. Until now, it was thought that specificity of such complexes was based essentially on intrinsic protein properties, but recently it was hypothesized in addition that lncRNA could also contribute. LncRNA have specific RNA sequences that recognize individual protein effectors leading eventually to ribonucleoprotein complexes that combine the regulative properties of both RNA and protein components. The idea of protein assembly dependent on a nucleotidic scaffold provides novel insights into therapeutic strategies to artificially modify specificity or activity of these bipartite signaling complexes.

#### 2.2.2. LncRNAs in Hematopoiesis

##### 2.2.2.1. LncRNAs in Erythropoiesis

The knowledge about involvement of lncRNA in hematopoietic differentiation or self-renewal of hematopoietic stem cells remains poor, and a part of the observed current results are derived from murine cellular models. However, unlike miRNAs, lncRNAs are very poorly conserved between species so that results cannot readily be extrapolated to human. However, it may be possible that the overall operating mode is preserved and that it would be possible to transpose a process from one species to another, considering that functions of lncRNA are mainly mediated by their secondary and tertiary structure and not only through their sequence [101]. Currently, such a transposition has yet to be demonstrated, but the importance of the secondary structures is shown by the joint action of human Alu, and mouse B2 that both show no direct primary sequence similarity but are both able to sequester RNA polymerase II [93,102,103].

In murine Ter-199 cells, a direct link between differentiation of hematopoietic cells towards the erythroid pathway and the lncRNA EPS (erythroid prosurvival) regulates inhibition of apoptosis during differentiation [104,105] and terminal erythropoiesis. Interference with the expression of this lncRNA (even at 50%) induced a significant decrease in cell proliferation and increased annexin V expression. At least one of the targets of lncRNA-EPS could be Pycard, which is an antiapoptotic gene whose protein product is able to activate caspases during apoptosis. Many examples of Pycard regulation by lncRNA-EPS exist. First of all, expression of Pycard and EPS are inversely correlated during erythropoiesis. Moreover, overexpression of EPS leads essentially to repression of Pycard (among those studied by the team of Hu *et al*.). In addition, Pycard overexpression generates the same phenotype than inhibition of EPS during erythroid terminal differentiation: inhibition of proliferation, induction of cell death and inhibition of enucleation, the final step of differentiation. Finally, overexpression of Pycard abrogates anti-apoptotic effects induced by ectopic expression of EPS.

We mentioned earlier possible interactions between miRNAs and lncRNAs, so lncRNAs could modulate miRNAs *via* their transcription or their sequestration, and conversely, a miRNA can regulate lncRNA indirectly by acting at least on translation of the TF involved in the expression of this lncRNA. A recent study focused on the interaction between lncRNA and miRNA, using Photoactivatable-Ribonucleoside-Enhanced Crosslinking and Immunoprecipitation (PAR-CLIP) and targeting Ago (Argonaute) proteins [92], the catalytic components of the RISC complex. Thus, the miRNA bound to Ago, guides it to its complementary sequence, allowing to identify the targets of miRNA and then to hypothesize the lncRNA involved in the action of the miRNA. This study mentioned four miRNAs targeting lncRNAs related to hematopoietic differentiation: miR-196A, miR-196B, miR-9-1 and miR-210. MiR196A and miR196B target HOXA5 [106] implicated in erythropoiesis and myelopoiesis [107,108]. MiR-9-1 and miR-210 [75] target ALK4, which can be related to erythropoiesis [109]. MiR-196A and B seem to interact with three ncRNAs referenced as ENST00000523790.1, ENST00000519935.1, ENST00000489695.1; miR-210 interacts with ENST00000525865.1 and miR-9-1 with ENST00000511014.1, ENST00000509783.1, ENST00000505030.1, ENST00000504246.1, and ENST00000500197.2. LncRNAs interact with miRNAs which themselves modulate genes involved in hematopoietic differentiation. This does not automatically mean that these lncRNA are involved in differentiation but could be targets for future investigations.

A better understanding of the interactions between miRNA and lncRNA will allow deducing the effects of an ncRNA when the effect of its interacting molecule is known. To simplify these analyses, the miRcode database was implemented [110], using GENCODE database and providing a comprehensive map of putative miRNA target sites across the GENCODE long non-coding transcriptome.

##### 2.2.2.2. LncRNAs in Hematopoietic Differentiation

H19 was one of the first imprinted noncoding RNAs to be identified [111,112]. It is transcribed from chromosome 11, contains the sequence of miR-675 in its first exon, and is one of the few lncRNAs conserved between species. H19 is co-regulated with insulin-like growth factor (IGF)-2, which is expressed from the same locus. In hematopoietic cells, this pair of growth regulatory genes is expressed in precursor cells and downregulated during normal and pathological differentiation [113]. However, expression of this ncRNA is not restricted to hematopoietic tissue, as it seems also expressed in ES cells, fetal and adult tissues [114]. Recent results show that H19 RNA could play a role in *trans* repression of Igf2 expression [115]. Abundant data about the implication of this lncRNA in cancer development show that H19 is essential for growth of some human tumor types, and it appears that its aberrant expression in cancer cells is due to modification of the methylation of imprinting control regions (ICR) of H19/IGF2 during differentiation. Recent data on bladder cancer documented how upregulated H19 increased cancer cell proliferation by increasing inhibitor of DNA binding 2 (ID2) expression [116].

In 2007, a study mentioned a new essential lncRNA implicated in hematopoietic differentiation [117]. Authors showed in several steps that a transcript is highly over-expressed during eosinophilic differentiation, and that the intronic region that encodes two transcript variants is highly conserved between human, mouse and chicken, which could reflect an evolutionary pressure leading to conservation of this potentially essential sequence. Moreover, the lack of a large ORF and paradoxically a poor amino acid conservation of small ORF strongly suggests that this transcript is an ncRNA. Results were confirmed by the lack of association of this transcript with ribosomes and absence of translation. An association with other types of proteins was proved nevertheless. This ncRNA was termed eosinophil Granule Ontogeny (EGO) and exists in two isoforms (EGO-A and EGO-B) of 535 and 1460 bases, respectively. Tissue-specific expression patterns suggest that EGOs act on bone marrow hematopoietic cell development but not on lymphoid development. Its silencing proved its requirement for major basic protein (MBP) and eosinophil derived neurotoxin (EDN) mRNA expression, but not for GATA-1. Its precise mechanism of action thus remains to be elucidated.

It has been shown recently in mice that HOXA6 and HOXA7 genes are indirectly regulated by lncRNA Mistral (MIRA) *via* recruitment of Histone-lysine *N*-methyltransferase (MLL)1 to chromatin, allowing transcription of HOXA6 and 7 genes. Results obtained in the murine system demonstrate involvement of ncRNAs in regulation of genes involved in mouse embryonic stem cell (mESC) differentiation [118]. These results can be extended beyond regulation of mESC, as HOXA6 plays an important role in the regulation of HSC self-renewal in human, and its overexpression is also involved in leukemia [119]; HOXA7 acts as an intermediate in the regulation of granulocytic differentiation repressed by the Polycomb group RING finger protein 2 (PCGF2) [120].

The transcription factor TAL1 (SCF) is a major regulator of hematopoietic differentiation [121]. The locus containing this gene also encodes two sense and antisense lncRNAs. Transcriptional inhibition of a lincRNA can lead to decreased, transcriptionally independent, expression of neighboring protein-coding genes in multiple human loci. Orom *et al.* showed that the lincRNA ENST00000444042.2 (CYP4A22-AS1-001) encoded by a sequence downstream of TAL1, on the same DNA strand, is a positive regulator of this gene’s expression [76]. The study shows that these effects are independent of lncRNA orientation towards their target sequences. Depletion of this lincRNA induces a specific, strong and significant decrease of TAL1 expression, but does not affect expression of other genes on the same locus. Depletion of ENST00000429328, a lincRNA located on the same locus, affects the expression of another closely located gene, deoxycytidylate kinase (CMPK1) but not the expression of TAL1. Possible mechanisms of action could be: interaction through sequence or structural homology with the encoding target gene, through the recruitment of transcriptional activator or basal TFs, or by the removal of a repressor. Finally, chromatin remodeling could also be involved.

Zhang *et al.* identified an intergenic transcriptional activity, located between the human HOXA1 and HOXA2 genes, which presents a myeloid-specific expression with specific up-regulation during granulocytic differentiation [122]. This lncRNA is called HOX antisense intergenic RNA myeloid 1 (HOTAIRM1). Its induction during RA-induced granulocytic differentiation acts through the RA receptor and depends on the expression of myeloid cell development factors targeted by RA signaling. Extinction of this gene attenuated the expression of HOXA1 and HOXA2 genes induced by RA, without affecting expression of more distal HOXA genes. This knock out also affects transcription of CD11b and CD18 involved in myeloid differentiation. Finally, authors suggest that HOTAIRM1 plays a role in myelopoiesis through modulation of HOXA cluster gene expression.

The lncRNA HOXA cluster antisense RNA 2 (HOXA-AS2) is transcribed from a gene located between genes HOXA3 and HOXA4, and is expressed in human peripheral blood neutrophils. This ncRNA plays an anti-apoptotic role in All trans RA (ATRA)-induced myeloid differentiation, protecting cells against ATRA-induced apoptosis probably through the inhibition of the TNFα-related apoptosis-inducing ligand (TRAIL) pathway [123]: in the promyelocytic leukemia cell model NB4, knockdown of HOXA-AS2 increases number of both apoptotic cells and TRAIL. Conversely, ATRA-induced NB4 cells treated with TRAIL show an increase in HOXA-AS2 expression.

The lncRNA Xist triggers X chromosome inactivation in female mammals and is expressed at an early embryonic stage. Xist is important for the development of mice embryos particularly between 3.5 and 12.5 days post-coitum. Moreover, the adults that already committed hematopoietic progenitors, rather than HSC, were also able to express this lncRNA [124]. Overexpression of Xist led to the loss of a majority of blood cell types besides pre-B and pre-T lymphocytes, which are the cell types able to reactivate Xist expression. It is interesting to note that this reactivation occurs in pre-B cells and pre-T lymphoid cells that undergo allelic exclusion of immunoglobulin heavy chain (*Igh*) or TCRβ loci. This observation led to the hypothesis of a link between allelic exclusion of antigen receptor genes in lymphocytes and X chromosome inactivation in embryonic cells.

A few years ago, a meta-analysis was conducted by Gibb *et al.* on 272 human serial analyses of gene expression (SAGE) involving lncRNA sequences. These analyses included 26 different normal and 19 cancer tissue types in order to establish a first global profiling of lncRNA [79]. Results show tissue-specific lncRNA expression in normal tissues and a systematic abnormal lncRNA expression in human cancer. The paper essentially discusses the difference between normal and cancerous tissues (breast, brain, lung and blood) and discusses lncRNA expression that could become interesting for future studies. Four lncRNA have an expression pattern specific to white blood cells: ENSG00000232192 (Transcribed ENST00000446321, 810 bases) also named RP11-62I21.1 is an lncRNA transcribed by the antisense strand of the protein-coding gene KIF26B-005, coding for the kinesin family member 26B which is not implicated in any hematopoietic differentiation. The second lncRNA gene, ENSG00000246100 (CTC-774J1.2), is located in an intergenic region and is transcribed to five lncRNA variants of 3322, 982, 504, 693 and 995 bases. ENSG00000227712 (RP11-418J17.3), transcribes into an lncRNA of 898 bases. The most interesting one, ENSG00000256910 (AL034397.1), corresponds to a sequence close to the gene encoding the protein Z39Ig associated with monocyte and macrophage cells and linked to inflammatory reaction [125,126]. The AL034397.1 gene produces two transcripts by alternative splicing containing respectively, two and three exons: ENST00000540516 and ENST00000538676. For both, the last exon contains the sequence of miRNA-223, cited previously for its essential implication in hematopoietic differentiation. No links have yet been established between these two non-coding RNAs, but it is tempting to speculate about an interaction between these two non-coding RNA.

Altogether there are less than a dozen lncRNAs with a confirmed involvement in hematopoietic differentiation. For some others, involvement may be assumed. It is not yet possible to define a network of global interactions between lncRNAs, but we strongly believe that such a regulatory network involving TFs, miRNA and lncRNA exists and will be defined in the near future.

## 3. Conclusions

Long term studies of TFs involved in the regulation of genes driving hematopoietic cell fate decision, proliferation, survival, differentiation and death, designed a complex regulation network from HSC to differentiated blood cells. During the last decade, an additional level of regulation was added to this TF network with the discovery and understanding of a new class of regulatory non-coding RNAs, the miRNAs. Understanding of functional and reciprocal interactions with TFs significantly improved our knowledge about the molecular biology of hematopoiesis and hematological diseases. Nowadays, the complexity of the regulatory network is even increasing through the emergence of lncRNAs as novel relevant regulatory elements in all biological processes. Even though the roles of lncRNAs remain partially undetermined, their ability to interact with miRNAs, regulatory proteins and DNA has been evidenced, including in the hematopoietic system. Together, TFs, miRNAs and lncRNAs most likely constitute a wide and complex regulatory network contributing to physiological hematopoietic development as well as to pathological alterations.
